# Action and object words are differentially anchored in the sensory motor system - A perspective on cognitive embodiment

**DOI:** 10.1038/s41598-018-24475-z

**Published:** 2018-04-26

**Authors:** Houpand Horoufchin, Danilo Bzdok, Giovanni Buccino, Anna M. Borghi, Ferdinand Binkofski

**Affiliations:** 1 Division for Clinical and Cognitive Sciences, Department of Neurology, RWTH Aachen University, Aachen, Germany; 2 Department of Psychiatry, Psychotherapy and Psychosomatics, RWTH Aachen University, Aachen, Germany; 3 Jülich Aachen Research Alliance JARA-BRAIN, Aachen, Germany; 4 Parietal Team, INRIA/Neurospin, Saclay, France; 5 Department of Medical and Surgical Sciences, University Magna Graecia, Catanzaro, Italy; 6 Department of Dynamic and Clinical Psychology, Sapienza University of Rome, Rome, Italy; 7 Institute of Cognitive Sciences and Technologies, Italian National Research Council, Rome, Italy; 8 Institute for Neuroscience and Medicine (INM-4), Research Center Jülich, Jülich, Germany

## Abstract

Embodied and grounded cognition theories have assumed that the sensorimotor system is causally involved in processing motor-related language content. Although a causal proof on a single-cell basis is ethically not possible today, the present fMRI study provides confirmation of this longstanding speculation, as far as it is possible with recent methods, employing a new computational approach. More specifically, we were looking for common activation of nouns and objects, and actions and verbs, representing the canonical and mirror neuron system, respectively. Using multivariate pattern analysis, a resulting linear classifier indeed successfully generalized from distinguishing actions from objects in pictures to distinguishing the respective verbs from nouns in written words. Further, these action-related pattern responses were detailed by recently introduced predictive pattern decomposition into the constituent activity atoms and their relative contributions. The findings support the concept of canonical neurons and mirror neurons implementing embodied processes with separate roles in distinguishing objects from actions, and nouns from verbs, respectively. This example of neuronal recycling processing algorithms is consistent with a multimodal brain signature of human action and object concepts. Embodied language theory is thus merged with actual neurobiological implementation.

## Introduction

Language as we know is unique to human beings. How it evolved is still a matter of thorough research. Embodied and grounded cognition theories state that higher cognitive processes such as language are implemented in neural substrates coding for the language content. Accordingly, language processing would re-enact sensorimotor, emotional, and introspective experience with words acting as tokens^1–6^. A number of behavioral and neuroscientific studies have recently demonstrated that manipulable nouns are grounded in perception and action systems (for reference see for example^7–14^). In the same vein, a lot of evidence has been provided of grounding of action verbs and sentences in the sensorimotor system (a non-exhaustive list includes^15–23^). In spite of these compelling demonstrations, to date a neurobiological explanation of the specific mechanisms underlying such grounding is missing. A major point of debate is how specific language elements, such as nouns and verbs, re-enact experiences of interaction with objects and actions^24^. A plausible neural basis was found with the discovery of mirror neurons and canonical neurons, both found originally in area F5 of the macaque monkey^25,26^. Canonical neurons are activated by plain sight of graspable objects and object directed actions^27^. Mirror neurons fire when a goal-specific action is performed, as well as when that same action is passively observed, for example grasping a cup to drink. Although research shows striking parallels between monkey and human brains, the level of integration of language content in sensorimotor systems remains unclear as single-cell recordings in humans are not ethical. Getting more insight into how deep semantics are grounded will give an important basis for treatment of stroke patients suffering from aphasia and apraxia. The hypothesis of the present study is that canonical neurons constitute the neural basis for representing objects and their linguistic counterparts, nouns. Correspondingly, mirror neurons would constitute the neural basis for actions and verbs. We expected the stimuli to recruit those visuomotor areas where canonical and mirror neurons were first described in humans^28^.

Although embodiment theories have emerged mostly in the 1990’s, and a lot further research has been done on this topic to date (for reviews, see^4,29–31^), especially the discovery of mirror neurons in macaque monkeys laid the groundwork for this line of research. One fMRI study^28^ e.g. showed that linguistic input leads to somatotopic activation of the motor and premotor cortex corresponding to either tongue, hand, or foot, respectively for lick, pick, or kick. Behavioral, transcranial magnetic stimulation (TMS) and electroencephalography (EEG) studies yield similar results (for an overview see^17,32^).

Because the conventionally used general linear model fMRI analyses with spatial smoothing discard the fine-grained spatial activity patterns that hold important information^33^, we used a multivariate pattern approach on non-smoothed data to increase sensitivity. Others suggested that sensory, cognitive, and motor processes manifest themselves as ‘neuronal population codes’^34^. In contrast, decoding models typically use learning algorithms for an informational agenda by showing generalization of robust patterns to new brain activity acquisitions^35–37^. This way, spatially distributed information can be effectively used^38,39^. Some brain-behavior associations might only emerge when simultaneously capturing neural activity in a group of voxels but disappear in single-voxel-based approaches, such as GLM analyses. As canonical and mirror neurons are believed to be intermingled in the brain, classical approaches with smoothing kernels of multiple millimeters could not capture their differential activations. Making use of linear support vector machines (SVM) and additionally a recently developed analysis method, namely predictive pattern decomposition^40^, the present study aims to bridge this gap.

Multivariate pattern analysis allows for the detection of subtle, distributed differences in brain activation properties by explicitly accounting for dependencies among voxels^38,41^. We formally tested for the existence of a distributed neural signature underlying “human action” as an instance of neural reuse^42,43^. Knops and colleagues^44^ could show that a classifier that was trained for evolutionarily conserved eye gaze processes was able to decode more recent mathematical calculation processes as a possible case of neural reuse in the human brain.

First, we aimed to show that observing objects and reading nouns recruit the same activation patterns. In parallel, observing actions and reading verbs also recruit the same related patterns. We therefore planned to test previous intuitions on grounding of words in the sensorimotor systems with a new method. Second, and more crucially, we aimed to disentangle the two systems during the processing of nouns and objects, as well as verbs and actions. If language is indeed grounded in the sensorimotor system, we predicted that nouns (ball, ring, cylinder) would re-enact the experience of interacting with objects. Verbs on the other hand should re-enact the experience of observing and performing object-directed actions. It is worth noting that the verbs we used refer to object directed actions (roll, catch, lift). More specifically, we hypothesized that the activation patterns common to nouns and objects and common to verbs and actions would recruit the canonical neurons system *(affordances)*^45^ and the mirror neuron system (MNS)^26^, respectively.

## Materials and Methods

The present investigation leveraged a toolbox of data-driven machine learning techniques that optimally allowed us to automatically extract useful neural patterns from fMRI recordings. First, support vector classification with recursive feature extraction in a predefined meta-analytic search space allowed for effective identification of subtle neural activity changes in the putative human MNS and in canonical neurons. Second, the ensuing findings were detailed by predictive pattern decomposition to uncover underlying components of variation that gave rise to the whole-brain activity in the different experimental conditions. As a neurobiologically informed topographical prior we benefitted from a previous coordinate-based meta-analysis^46^. This quantitative synthesis isolated the consistent neural activity increases during a variety of experimental tasks on “action observation” and “action imitation” – two cognitive processes with close relationship to the putative mirror neuron system. Functional MRI data was acquired from healthy participants. The data was then analyzed using Recursive feature extraction (RFE) that was applied on the data using a mask obtained from an ALE meta-analysis^46^. This way the most relevant voxels could be extracted. To demonstrate the neural recycling effect, a 2-fold cross-validation scheme was used: The training data consisted of the fMRI neural activity maps acquired during verb and noun trials from all subjects, while the testing data composed these trial-wise fMRI maps from the objects and objects-with-hand conditions. As such, we trained the classification algorithm on one subset of the experimental conditions and evaluated the fitted classification algorithm on unseen, statistically independent brain images from the remaining conditions. We then added additional statistical analyses to corroborate the results. Following, a predictive pattern analysis was conducted, extracting ten whole brain activation patterns (components).

### Participants

Twenty healthy native German speaking participants (10 female; mean age 24.4 years; SD 3.14; range, 18–31 years) participated in this study, receiving monetary reward for participation. All participants were right-handed according to the Edinburgh Handedness Inventory^47^ (mean score 92.4; SD 8.8) and reported normal or corrected-to-normal vision. The study was approved by the local Institutional Review Board of the Medical Faculty of the RWTH Aachen University and was conducted according to the Convention of Helsinki. Written informed consent was obtained from all participants prior to testing.

### Stimuli

**Figure 1:**
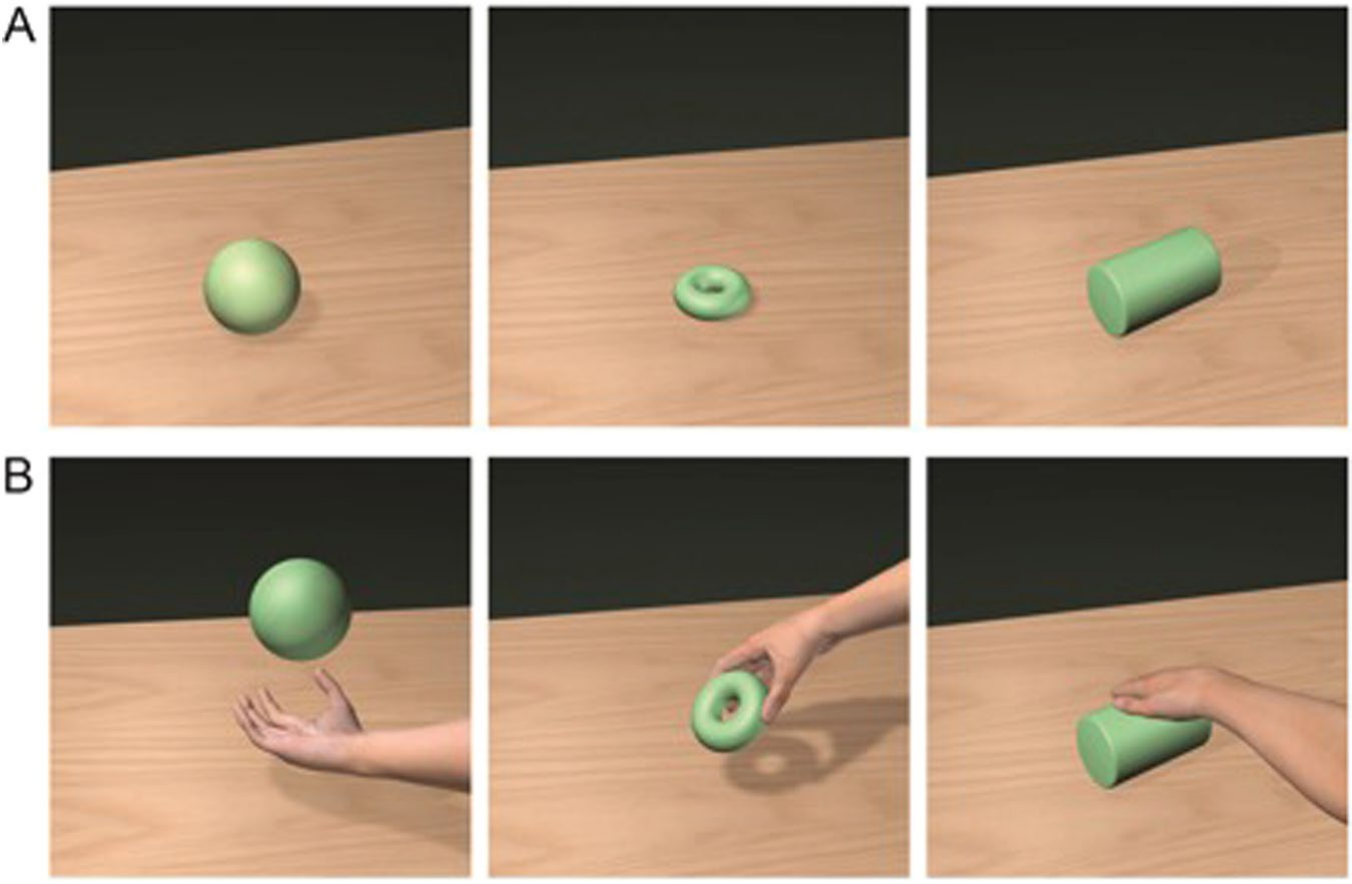
Stimuli and experimental paradigm. (**A**) shows stimuli for condition “object”, (**B**) depicts “action”. The corresponding textual stimuli were written in white letters on a black background and presented centrally (“Kugel”, “Ring”, “Zylinder” for nouns, and “fangen”, “nehmen”, “rollen” for verbs). Stimuli were presented in a randomized fashion. Each trial started with the presentation of a fixation-cross in the center of the screen for 1000 ms, followed by the stimulus for a further 1000 ms. The stimulus was followed by a random jitter of 800–1000 ms and a blank inter-trial interval of 7200 ms.

The experiment consisted of four sessions with four conditions and three stimuli for each condition. In the first condition, named Objects (from here on referred to as “O”), participants were presented with 3-dimensional geometrical objects. The three stimuli were a ball, a cube, or a cylinder. The second condition, Implied Actions (IA), showed the same objects as the “O” condition but with the addition of a hand acting on them, implying an action. The three implied actions were rolling, catching or lifting. Stimuli were chosen to be similar to those used in monkey studies where mirror neurons were discovered^48,49^. The distinctiveness of the objects was assured by a preceding poll (n = 20), which showed that at least 80% of the sample agreed on the pictures’ meaning. The third and fourth condition showed the written names of the objects (nouns) and implied actions (verbs) in white letters on a black background (Fig. 1). As the used verbs and nouns are high frequency words, the differences between verbs and nouns should have been clear to the participants. To avoid possible associative learning effects, stimuli were never shown at the same time.

### Procedure

All participants received an introduction to the task and the stimuli outside of the scanner. All stimuli were presented to all participants in a randomized fashion in each of the four runs. The experiment was programmed with the Presentation software package (Neurobehavioral Systems, Albany, CA, USA).

Each trial started with the presentation of a fixation-cross in the center of the screen for 1000 ms, followed by the stimulus for 1000 ms. The stimulus was followed by a random jitter of 800–1000 ms and a blank inter-trial interval of 7200 ms. Each block consisted of 60 experimental trials (12 stimuli, 6 pictorial, 6 textual, each individual stimulus presented a total of 5 times) plus 4 oddball-trials, which were added to keep participants’ attention, with a 20 second rest-condition after 32 trials. To ensure attentiveness, participants were required to press a button with their right index finger whenever they saw a red object or read a pseudo word. These trials were excluded from the analysis. In the scanner, visual stimuli were presented via MRI compatible 3D goggles (VisuaStim XGA, Resonance Technology) with a horizontal viewing angle of 30° and a vertical viewing angle of 22.5°. Data recording and stimulus presentation were accomplished using Presentation (Neurobehavioral Systems, Albany, CA, USA).

### MRI Image acquisition

Imaging was performed on a SIEMENS 3 T magnetic resonance scanner using an 8-channel head coil. To minimize head movement subject’s heads were stabilized with foam cushions. Participants also used foam earplugs for noise protection and noise-reducing headphones.

For fMR imaging we used a T2*- weighted echo planar imaging (EPI) sequence (1600 ms repetition time, 30 ms echo time, 67° flip angle, interleaved, 64 × 64-pixel acquisition and reconstruction matrix, 19.2 cm field of view, 26 slices), with a resulting voxel-size of 3.5 × 3.5 × 3.85 mm and 540 dynamic scans per run, resulting in a total of 2160 scans for the whole experiment. T1-weighted anatomical images were also required for all participants subsequent to the functional scans with an MPRAGE sequence (TR = 1900 ms, TE = 2.52 ms, FA = 9°, FOV = 256 × 256, ST = 1 mm, spatial resolution 0.98 mm × 0.98 mm × 1 mm).

### Preprocessing and Image preparation

Images were analyzed with SPM10 software (http://www.fil.ion.ucl.ac.uk/spm/). The first 5 scans of each participant were removed to allow for full T1 saturation, the remaining images were slice timing corrected, realigned to mean EPI image. Subsequently the data was co-registered to the anatomical images and normalized to the Montreal Neurological Institute (MNI; (Montreal Neurological Institute; http://www.mni.mcgill.ca/) reference brain. No spatial smoothing was applied for the following statistical analysis. Data was then realigned and resliced to the mean image. Following, every single trial for every subject was modelled as a single condition to allow each stimulus to be used in the MVPA. This resulted in 60 individual modeled trials per subject resulting in 1200 trials in total.

### Multivariate Pattern Analysis (MVPA)

**Figure 2:**
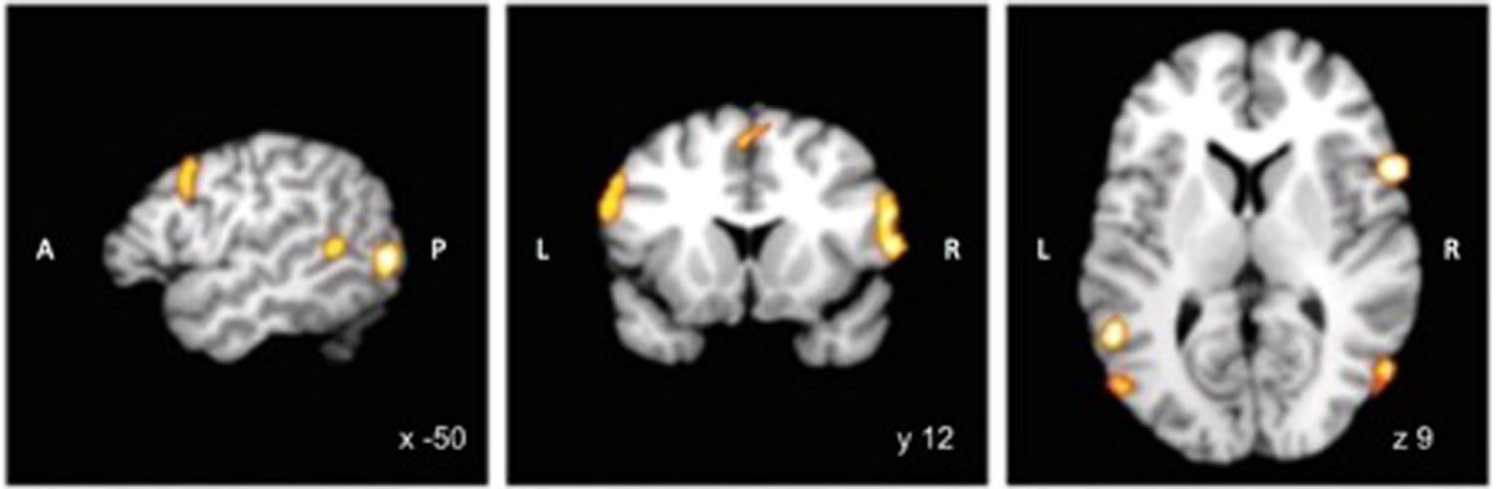
Mask used for Recursive Feature Extraction. The mask was created by a conjunction of “observation” and “imitation” results, obtained from the results of a meta-analysis^46^; coordinates are in Montreal Neurological Institute space. Abbreviations: A: anterior, P: posterior, L: left, R: right.

During scanning, participants were exposed to four categories of visual stimuli. Pictorial stimuli depicting (1) plain objects and (2) objects with hand-object interactions, and the corresponding words (3) nouns and (4) verbs. A linear classifier, i.e. a SVM, was trained on half the 4800 neural activity maps, thus 2400 examples of training data, neural activity maps from 1200 experimental trials with plain textual stimuli with verbs versus nouns. Each experimental trial consisted of four neural activity maps, due to the long trial duration. That means each ‘trial’ corresponds to one fMRI whole-brain image and one stimulus presentation. The trained binary classification algorithm (SVM) was then tested on the other 2400 independent experimental trials, thus 2400 example as testing data, with more complex geometrical 3D objects in two conditions: plain objects and hand-object interactions. We based our definition of the MNS on the neuroanatomical structures identified in a previously published quantitative meta-analysis^46^ (see Fig. 2 and Table 1). The training data comprised the beta-estimate maps from each experimental trial presenting written stimuli with verbs or nouns, while the independent test data comprised trials with pictorial stimuli with objects. The training set and test set were standardized using mean centering and unit-variance scaling. This data transformation compensates for a possible wider variation in signal amplitude in some voxels than in others^50^. A self-learning algorithm (i.e. linear support vector machine) was fitted to classify noun versus verb conditions from 2,400 neural activity maps. All present multivariate analyses were conducted in an across subject context. This SVM predicted the class of each neural activity map (i.e., action or no action), based on a linear combination of the weighted features. The trained linear classification algorithm was then applied to the non-overlapping experimental conditions with complex geometrical 3D objects with or without action-implying hands. The linear classifier designated each of the 2,400 test maps as action or non-action. Mean averaging across prediction instances yielded out-of-sample performance and binomial-tested p-values (cf.,^50,51^).

**Table 1:** Peak MNI coordinates and cluster size for meta-analytic mask (left) and for results of recursive feature extraction (RFE) (right) with their corresponding anatomical structures.

Cluster	Mask^a^				RFE result^b^				
	Cluster size	Peak MNI coordinates			Cluster size	Peak MNI Coordinates			Macroanantomical location
		*x*	*Y*	*z*		*x*	*y*	*z*	
1	275	56	13	18	41	55	13	18	Right BA 44, BA 45
2	229	−54	7	33	27	−54	7	35	Left BA 44, pars opercularis
3	174	53	−65	4	22	53	−66	4	Right MTG/FFG
4	141	−37	−40	50	19	−37	−40	50	Left IPL/aSMG
5	130	−50	−70	4	17	−51	−70	4	Left MTG
6	109	45	−57	−17	15	−53	−50	9	Left MTG
7	91	−53	−50	9	11	44	−58	−15	Right FFG/ITG
8	36	0	13	53	6	52	−36	48	Left and right SMA
9	33	51	−36	49	4	1	14	52	Right SMA

Abbreviations: Brodmann Area (BA); Middle Temporal Gyrus (MTG); Fusiform Gyrus (FFG); Inferior Parietal Lobule (IPL); Inferior Temporal Gyrus (ITG); Supplementary Motor Area (SMA), anterior Supramarginal Gyrus (aSMG).^a^see Fig. 2; ^b^see Fig. 3.

Dimensionality reduction was applied to enable statistical tractability and improve functional specificity. First, the gray-matter voxel space was reduced to a meta-analytical definition of the mirror-neuron system. This was obtained by all non-zero voxels of the AND-conjunction between “action observation” and “activation imitation”^46^. Thus, the feature space input to the classification algorithm only considered neural activity information from brain voxels that have been significantly associated with many previously published studies on observation and imitation activation. Second, the optimal number of predictive voxels within the meta-analytically constrained brain space was identified by recursive feature extraction. In every step, the number of relevant voxel features was reduced by 50% to repeat model fitting and prediction with more parsimonious activity information. Importantly, the most discriminatory voxels were chosen from the training set alone and used for feature selection in the test set. In this way, we could formally test whether a classifier can exploit multivariate neural activity in mirror-neuron regions to generalize to two unseen experimental conditions, which imply action and the lack thereof.

### Predictive pattern decomposition

The feasibility of combined representation learning and pattern classification has recently been demonstrated by introducing a novel statistical model for multi-task prediction based on brain activity maps^40^. Semi-supervised factored logistic regression is an equally weighted composite model of an exploratory auto-encoder module and an inferential task prediction module by logistic regression (lambda = 0.5). The auto-encoder, a generalization of independent component analysis or principal component analysis^52^, discovers the most important spatiotemporally coherent components in the neural activity maps across experimental tasks. The factored logistic regression simultaneously projects the activity maps onto these emerging components to predict the experimental tasks from relative component implications. The linked optimization goals of data representation into component sets and task prediction based on components result in the set of distributed activity patterns that are most pertinent in neurobiological terms in explaining each experimental task. Three additional penalty terms were added to the optimization goal. L1-penalization encouraged automatic variable selection (i.e., setting component weights to exactly zero) and L2-penalization encouraged variable shrinkage (i.e., privileging small component weights), amounting to Elastic-Net-type model regularization. Finally, an orthogonality penalty encouraged shrinkage in the off-diagonal entries of the component covariance matrix (i.e., maximal differences between each pair of components). The ensuing compound optimization objective was numerically approximated by RMSProp solvers (learning rate = 0.0001, maximal epochs = 500, decay rate rho = 0.9, global damping factor epsilon = 0.000001, gradient clipping), a modern variant of stochastic gradient descent^53^. All model weights were initialized by Gaussian random values multiplied by 0.004 (i.e., gain), and bias parameters were initialized to 0.

In this way, we have blended representation modeling and task classification into a unified statistical modeling approach for the discovery of the set of unknown functional compartments that are most predictive for mirror-neuron phenomena in the brain.

On completion of voxel-based classification, we recast the correspondence between mirror-neuron function and brain activity as a representation problem^54^. The current literature supports the notion that a particular mental operation is probably realized *between the two extremes of functional segregation into specialized brain regions and intertwined brain networks*^55–58^. This analysis therefore originates from the assumption that the optimal set of brain regions, brain networks, or other functional units underlying the mirror-neuron systems has yet to be discovered. We performed a decomposition of the task fMRI data into a set of components with spatiotemporally coherent neural activity, whose combinations allow for the best classification of the four mirror-neuron-related experimental tasks. Instead of assuming the neurobiological validity of voxel units, this analysis serves to discover those distributed voxel patterns, whether closer to region or to network notions, that are most predictive for mirror-neuron processes. All maps were resampled to a common 60 × 72 × 60 space of 3 mm isotropic voxels and gray-matter masked (at least 10% tissue probability), with each task map transformed into 79,941 voxels of interest representing z-values in gray matter.

### Software implementation

All statistical-learning analyses were performed in Python. Scikit-learn^59^ provided efficient, unit-tested implementations of state-of-the-art statistical learning algorithms (http://scikit-learn.org). This general-purpose machine-learning library was interfaced with the neuroimaging-specific nilearn library^60^ for high-dimensional neuroimaging datasets (http://github.com/nilearn/nilearn). Theano was used for automatic, numerically stable differentiation of symbolic computation graphs^61,62^. All Python scripts that generated the results are accessible online for reproducibility and reuse (https://github.com/banilo/horoufchin2017scirep).

### Significance Statement

Embodiment Theories are increasingly influential and many studies point to a connection between language and the sensorimotor system^7–23^. However, the level of integration of language in the sensorimotor systems was left unclear as single-cell recordings in humans are rarely ethical. Employing a new approach to analyze fMRI data, the present results are the first to show a clearer link on a neural level between the sensorimotor system and language systems in humans. By doing this, we could demonstrate common neural activity patterns between objects and nouns, and actions and verbs.

## Results

### Multivariate Pattern Analysis (MVPA) Results

**Figure 3:**
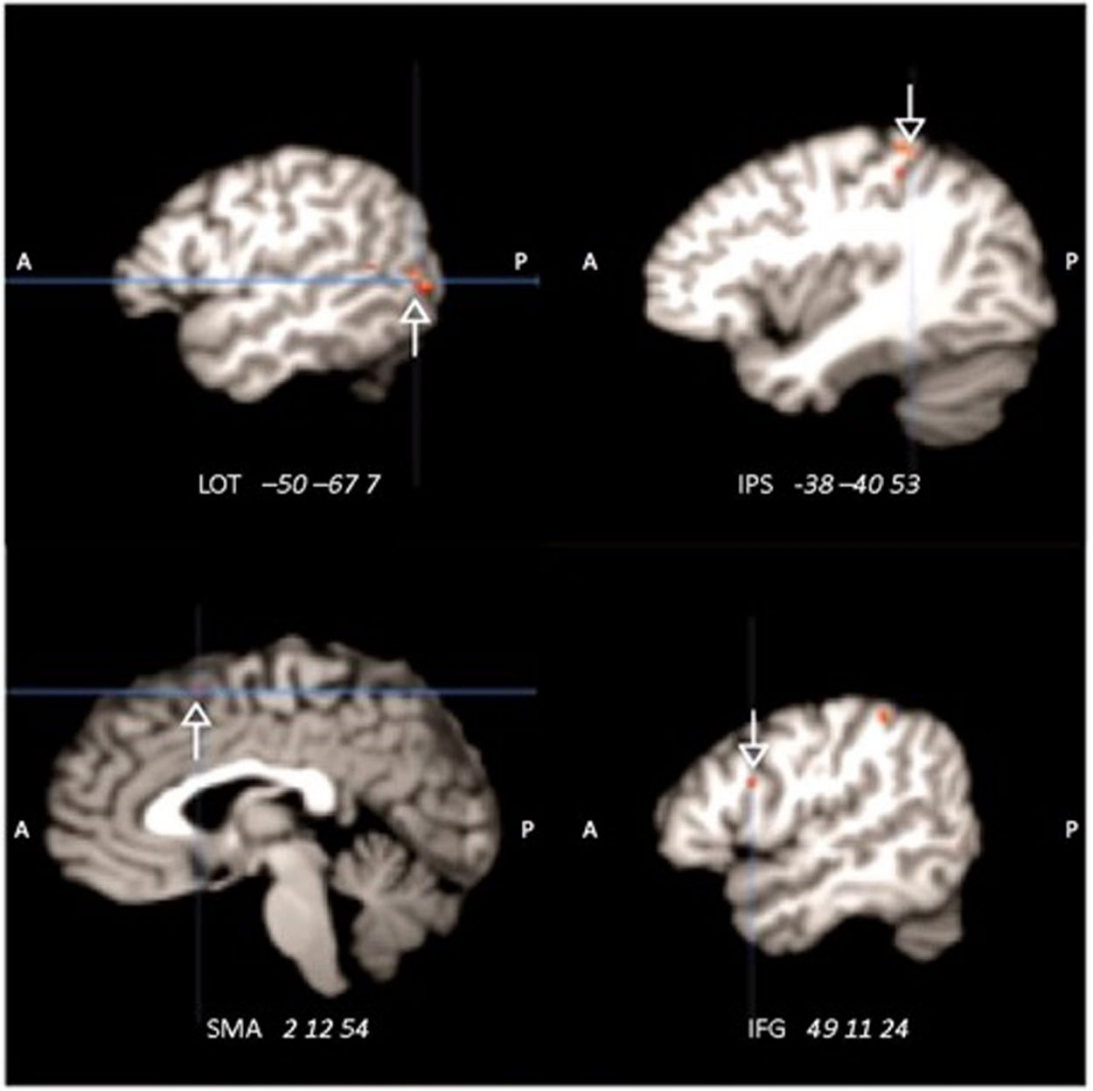
Neural activity pattern of multimodal action signature. This depicts the model weights underlying action-versus-object distinction that successfully generalized across diverse stimulus materials. Results of recursive feature extraction (RFE). Coordinates are in Montreal Neurological Institute space. Abbreviations: A: Anterior, P: posterior, LOT: lateral occipito-temporal cortex, IPS: intraparietal sulcus, SMA: Supplementary motor area, IFG: inferior frontal gyrus.

For the MVPA analysis the search space was constrained to the afore-mentioned meta-analytically defined search volume underlying action observation and execution (bilateral inferior frontal gyrus (IFG), Inferior parietal sulcus (IPS), MT/V5), the most discriminatory neuronal populations were automatically identified by recursive feature extraction (Fig. 3). The classifier successfully exploited multivariate activity patterns in candidate mirror neuron regions to generalize to two unseen trials from experimental conditions with different pictorial stimuli (54% out-of-sample performance, p < 0.000186, chance level at 50%). In absolute numbers, the hits and failures in concrete numbers in the original analysis were 1292 correct and 1108 wrong predictions in a total of 2400 experimental trials with stimuli of different modality. This effect is comparable to previous neural recycling studies^44^. Nevertheless, we have obtained confirmation in a set of three supplementary analyses: i) A jackknife analysis resulted in a mean accuracy of 53.62 +/− 0.44% (mean p-value 0.0015 +/− 0.0022), i.e. we achieve a p-value < 0.002 on average when always leaving all data from one subject out. ii) A bootstrapping analysis with 100 bootstrapping replications resulted in a mean accuracy of 53.06 +/− 1.07% (mean p-value 0.028 +/− 0.06), i.e. p < 0.05 across 100 bootstrapping iterations. iii) Using a split half analysis with 100 iterations, the mean accuracy was still above chance when only considered random halves of the data with an average accuracy of 52.88 +/− 1.26%, despite the considerably lower statistical power due to the much fewer training examples. Taken together, across three different types of perturbations of the brain data the identified predictive patterns successfully extrapolated to the new task comparison.

In short, a pattern recognition algorithm trained to distinguish nouns from verbs can readily discriminate object pictures from actions.

### Predictive pattern decomposition Results

**Figure 4:**
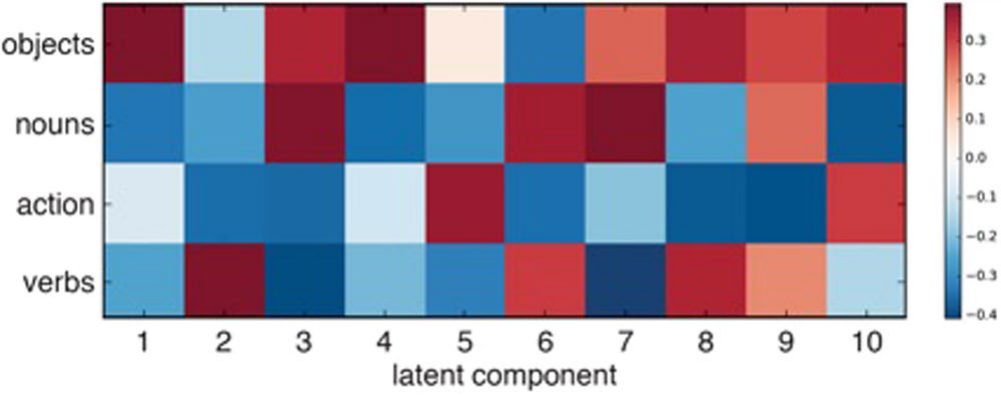
Results of the predictive pattern decomposition. Relative contributions of each of the ten most predictive patterns are shown for each of the stimulus conditions. Latent components describe the weight each pattern of activation adds to each of the categories on the y-axis. Component 3 for example shows common activation for objects and nouns, and common decrease in activation for action and verbs. Note that the plus and minus signs are coincidence, i.e. actions and verbs, and objects and nouns, are connected equally in this component, respectively.

**Figure 5:**
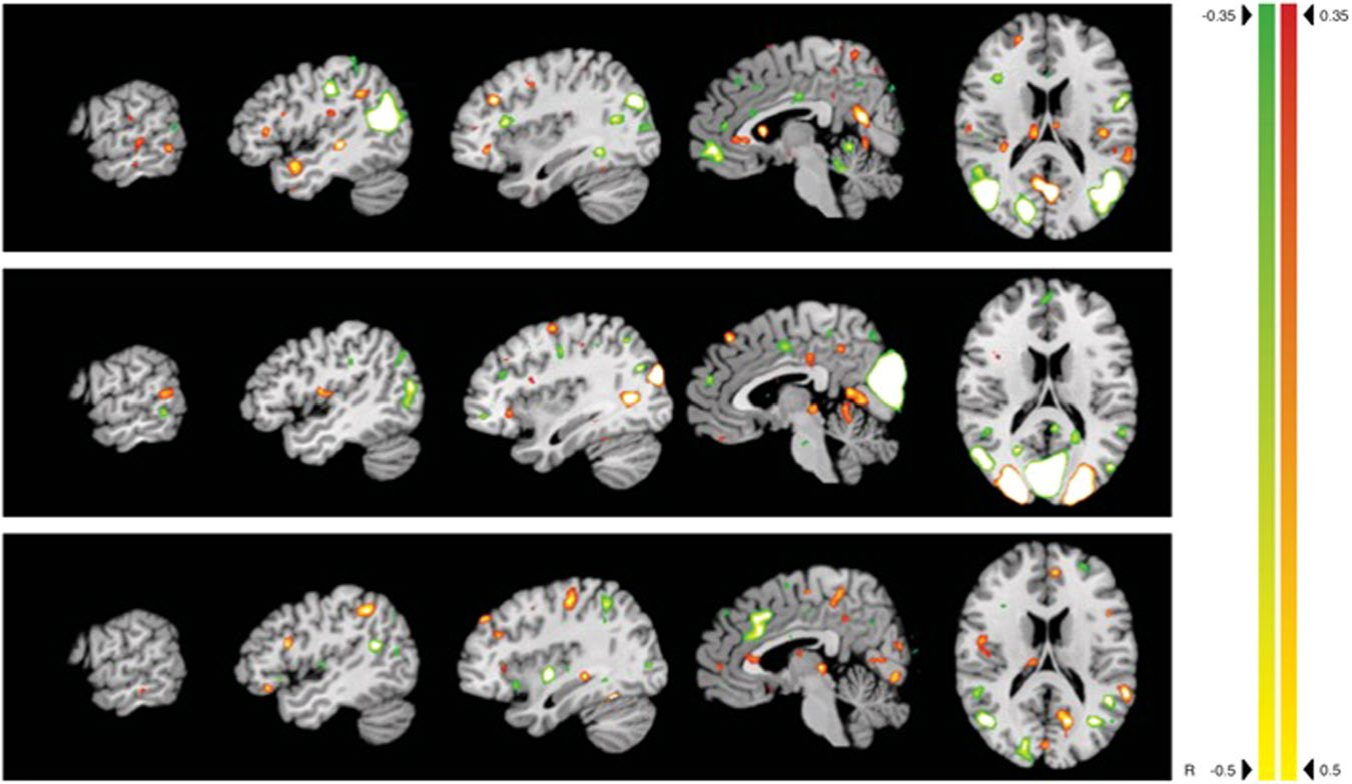
Activations and  deactivations for component 3, 6, and 7 (from top to bottom). Slices at x −63, 47, −37, −4, and z −16, respectively. Color bars indicate corresponding *z*-values.

**Figure 6:**
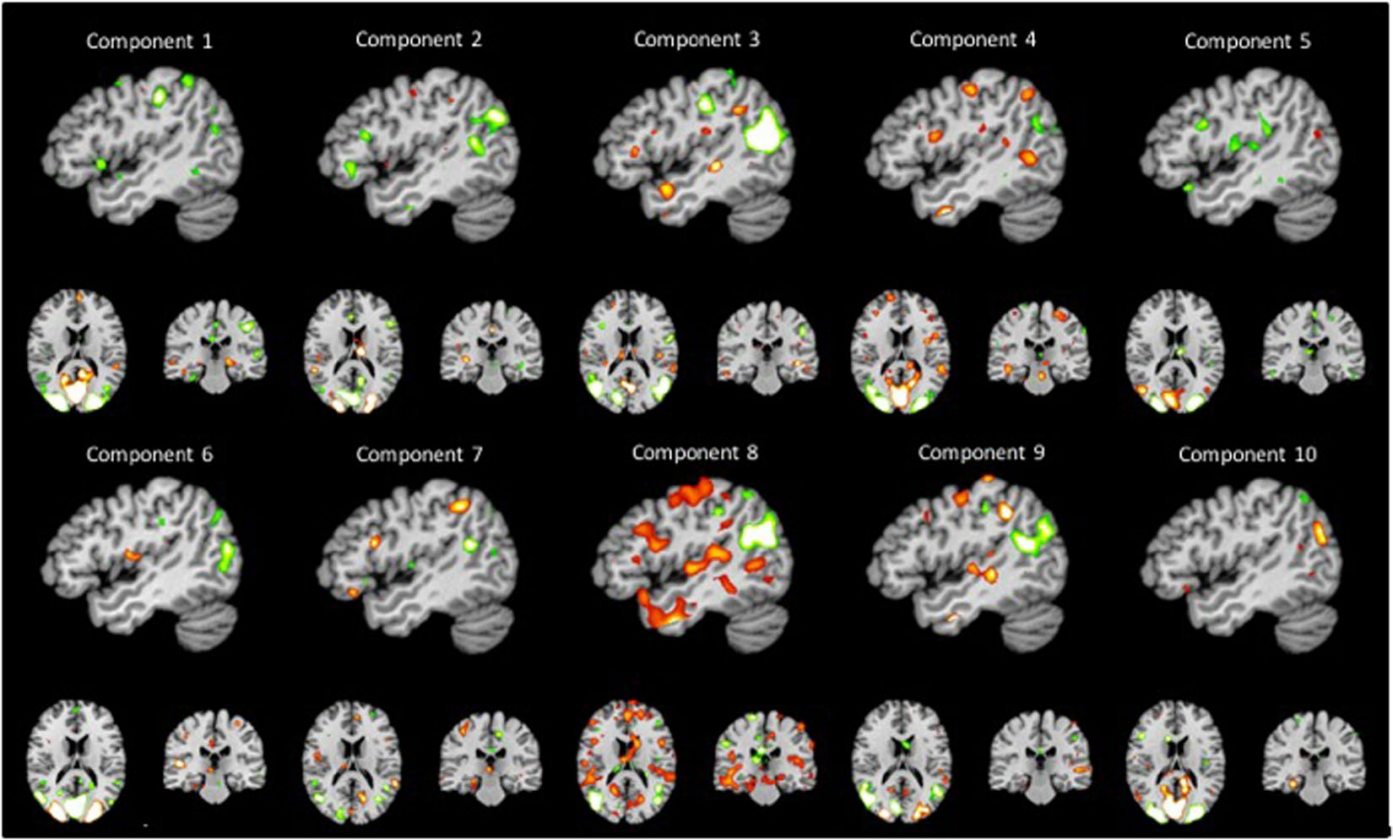
Overview of all 10 components extracted by predictive pattern decomposition. Red shows activation, green deactivation. x at 4.

The multimodal nature of these structured neural encodings for human action was further computationally dissected by a novel machine-learning method^40^. Predictive pattern decomposition was used to obtain the ten most predictive whole brain patterns that consist of latent components (Figs 4–6), each of which contained discriminative elements to distinguish between the four experimental conditions. Importantly, this statistical tool extended existing latent factor models by also estimating the relative contributions of the discovered multivariate patterns to the four experimental conditions to be predicted. The generalization of action specific processing patterns in the MNS is thus complemented by formally isolating object specific processing patterns in the canonical neurons.

Component 3 shows a pattern of strong activation of IFG (BA 44 and BA 45), supplementary motor area (SMA), BA 18 (LOTC), BA 21, caudate nucleus, and right parahippocampal activation (Fig. 5). These areas have previously been described in either object processing^7,63,64^ or noun processing^65^. Supporting the hypothesis of the current study, component 3 exhibits a coherent activity *decrease* during action-related experimental conditions and in verb processing in the ventral and dorsal medial prefrontal cortex, extending into the anterior cingulate cortex, posterior cingulate cortex / precuneus, bilateral temporo-parietal junction, right temporal pole and left middle temporal gyrus.

Component 6 was activated by both word categories. It showed strong activation of Brodmann Area (BA) 44, medio-temporal gyrus (MTG), left lateral occipito-temporal cortex (LOTC), and right caudate, superior middle occipital cortex bilaterally and retrosplenial activation, further activation of Heschl’s gyrus (Fig. 5). These areas are known to be strongly involved in language processing^66^.

Also, activations of for component 7 are known to be involved in language processing but also objects processing. It showed activation of right IFG (BA 44 and BA 45), parietal/postcentral areas, middle frontal gyrus, fusiform gyrus, cuneus, thalamus, and putamen (Fig. 5). All the afore-mentioned areas are involved in object processing, including recognition and naming of objects.

## Discussion

The aim of our study was to use the MVPA and the predictive pattern decomposition analysis techniques to find out whether a common neural activation pattern exists in the human brain for nouns and objects, or verbs and actions, respectively. Further on we hypothesized that the common representation of actions and verbs would activate the human homologue of the mirror neuron system (MNS) and the representation of objects and nouns would activate the human canonical neurons. The two groups of neuronal populations, i.e. MNS and canonical neuron system, are believed to be intermingled or in very close proximity to each other in the human parietal and premotor areas. To date, disentanglement of differential activity responses with conventional fMRI analyses has not been achieved. The present fMRI study aimed to fill this gap.

The results of the MVPA indeed showed that a linear SVM which was trained on words to discriminate verbs from nouns could successfully generalize to also categorize pictorial stimuli of actions from objects. Furthermore, as hypothesized the voxels holding the most information for this categorization were found in areas commonly referred to as human homologues for the mirror neuron system^46^, as it has been originally found in macaque monkeys. Based on the afore-mentioned findings, we believe we have disentangled neural activations of the adjacent canonical and mirror neuron systems by breaking them into overlapping but independent activation patterns. Each of these systems provides unique contributions to the processing of nouns versus objects, and verbs versus actions. To confirm this new approach, we took a closer look at each component, restricting ourselves to the description of the most relevant components. The inspection of component 3 provides the best confirmation of our hypothesis. On the one hand, it contains an expression of similar activation patterns for objects and nouns and on the other hand, it shows a pattern of common decreased neural activation for actions and verbs.

The areas activated for objects and nouns include, among others, the anterior temporal cortex which plays a pivotal role in processing semantic knowledge for objects and words^67^, and the posterior IPS which is involved in processing of object properties^68,69^.

The areas that show a common decrease in activation for actions and verbs include the inferior frontal gyrus as well as the AIP, which are in accordance with our understanding of MNS localization in the human brain^46^.

The activations for objects and nouns, and the decreases in activation for actions and verbs thus are co-localized (see Fig. 5) in the premotor cortex as well as the parietal cortex, further corroborating the assumption that in humans the mirror neuron system and the canonical system are in close proximity to each other.

To substantiate the results of the predictive pattern decomposition, we looked closer at component 6, which is activated for both verbal stimuli, and component 7, which is primarily related to object and noun processing. Component 6 shows activation in the IFG (Broca’s region), IPL, MTG, MFG, and Heschl’s gyrus, all consistent with typical language processing areas^65,70–72^. Component 7 on the other hand shows activation in IFG, MTL, IPL, and the fusiform gyrus, i.e. areas that are involved in object processing as well as word processing (here nouns)^73–75^. The resulting activations further confirm our approach.

For the component 3 we also found interesting additional activations in a set of regions (ventral and dorsal medial prefrontal cortex, extending into the anterior cingulate cortex, posterior cingulate cortex / precuneus, bilateral temporo-parietal junction, right temporal pole and left middle temporal gyrus), collectively known as the default-mode network (DMN). DMN was found to drive task-specific activity patterns when external cues indicate any form of action, but to decrease in the absence of action stimuli. It indeed has been proposed that the DMN constantly generates predictions of potential future events based on extracted environmental regularities^76,77^. This contention also dovetails with a possible role in constructing probabilistic mental scenes that influence ongoing decision-making by estimating behavioral outcome schedules^78,79^.

We would also like to discuss our data with respect to neuronal recycling theories^42,43,80^. The concepts of these theories state that preexisting neural systems can be re-used for more novel tasks. One example for this is reading, an evolutionary novel skill that is not innate but has to be learned. It is argued that these cultural inventions are embedded in neural niches that fit the required functions and become specialized after extensive training. The present results fit in with these theories, that somatosensory systems are re-used for further language and reading development. While we do not claim that all language must be embedded in the somatosensory systems, we argue that the present results show that at least specific action verbs seem to be represented in the somatosensory systems, more precisely the human homologue of the mirror neuron system originally found in macaque monkeys. The fact that the same methods show similar results for objects and their corresponding words, adds further support for this theory. The re-used neural structures used for this category of words fit the canonical neurons, also found to be localized adjacent to the mirror neuron system, not activated by action but by plain sight of manipulable objects.

In a nutshell, behavioral studies have suggested that nouns and objects have common underlying neural mechanisms^12,81–83^. The present fMRI study shows a clear overlap of brain areas and similarities of patterns during the processing of objects and nouns.

More broadly, predictive pattern decomposition hence identifies a potential coupling mechanism between the two large-scale systems of MNS, canonical neuron system, and DMN during action perception. The present findings therefore complement the region- and network-centric interpretations in the existing literature with an invigorating view of functional brain organization based on dynamic network reconfiguration^55^.

## Conclusion

Taken together, our findings from combined computational and experimental approaches strongly support the hypothesis that functionally mirror neurons and canonical neurons act in parallel and in very close anatomical proximity. Further, these results confirm the predictions of embodied and grounded cognition theories. Based on neural recycling theories, which are long embraced by the experimental psychology communities, our results demonstrate that words, such as verbs and nouns, are grounded in the sensorimotor system (see Component 6), and that they activate the canonical and mirror neuron systems in subtly different ways. Specifically, the common activation patterns of objects and nouns (Component 3 and 7) and of actions and verbs (see Fig. 6) engage brain areas typically associated with the canonical and the mirror neuron systems, respectively. Thus, we can state that our results show that the two grammatical classes nouns and verbs activate the canonical or the mirror neuron system which indicates that each has a distinct functional organization. At the same time however, the overlapping activation areas suggest that our semantic system is highly integrated and distributed. In conclusion, verbs and implied actions recruit the same cognitive networks that allow us to interact with objects and individuals in everyday life.
